# Tobacco‐Attributable Burden of Early‐Onset Ischemic Heart Disease From 1990 to 2023: A Global Analysis Using GBD 2023 and Age‐Period‐Cohort Modeling

**DOI:** 10.1002/clc.70451

**Published:** 2026-08-24

**Authors:** Xin Ouyang, Xiangting Chen, Fei He, Qingbin Li, Xuhui Lin, Kailun Qiu, Ruowen Zhao, Bowen Liu, Yibin Liu, Pengcheng He

**Affiliations:** ^1^ Guangdong Cardiovascular Institute, Guangdong Provincial People's Hospital Guangdong Academy of Medical Sciences Guangzhou China; ^2^ Department of Ophthalmology, Sun Yat‐Sen Memorial Hospital Sun Yat‐Sen University Guangzhou China; ^3^ Department of Cardiology, Guangdong Provincial People's Hospital, Guangdong Academy of Medical Sciences Southern Medical University Guangzhou China; ^4^ Department of Cardiology The First Affiliated Hospital of Sun Yat‐Sen University Guangzhou China; ^5^ Institute of Future Health South China University of Technology Guangzhou China

**Keywords:** age‐period‐cohort analysis, Bayesian projection, early‐onset ischemic heart disease, global burden of disease, tobacco

## Abstract

**Background:**

Early‐onset ischemic heart disease (IHD) is a growing global threat, with tobacco exposure as a key modifiable risk factor. A global assessment of its tobacco‐attributable burden is needed.

**Methods:**

Using Global Burden of Disease Study 2023 data, we assessed tobacco‐attributable deaths and disability‐adjusted life years (DALYs) from early‐onset IHD in 204 countries and territories during 1990–2023. Trends were evaluated using estimated and average annual percentage changes (EAPCs and AAPCs). Age‐period‐cohort (APC) models characterized age, period, and cohort patterns, and Bayesian APC models projected age‐standardized rates to 2050. A sensitivity analysis compared the sexes aged 40–54 years.

**Results:**

From 1990 to 2023, tobacco‐attributable DALYs increased from 15.21 to 17.12 million and deaths from 342 900 to 386 300. Conversely, ASDR declined from 381.54 to 221.82 per 100 000 (EAPC −1.84%, 95% CI −1.94 to −1.73) and ASMR from 8.69 to 4.96 per 100 000 (EAPC −1.88%, 95% CI −1.98 to −1.77). Male rates remained higher, including when both sexes were restricted to ages 40–54 years. APC analyses showed significant favorable net drifts and lower period and cohort rate ratios in recent periods and birth cohorts. Bayesian projections suggested declining ASDRs through 2050, whereas ASMR estimates were stable or slightly increased with widening uncertainty.

**Conclusion:**

The age‐standardized burden of tobacco‐attributable early‐onset IHD declined substantially between 1990 and 2023, but absolute DALYs and deaths increased and disparities by development level and sex persisted. Stronger tobacco control and integrated cardiovascular prevention are needed, particularly for high‐risk males and in low‐ and middle‐SDI settings.

## Graphical abstract

Using GBD 2023 data, we found that age‐standardized tobacco‐attributable early‐onset ischemic heart disease burden declined globally from 1990 to 2023, while absolute DALYs and deaths increased. Persistent male and regional disparities, together with uncertain mortality projections, underscore the need for stronger tobacco control and integrated cardiovascular prevention.

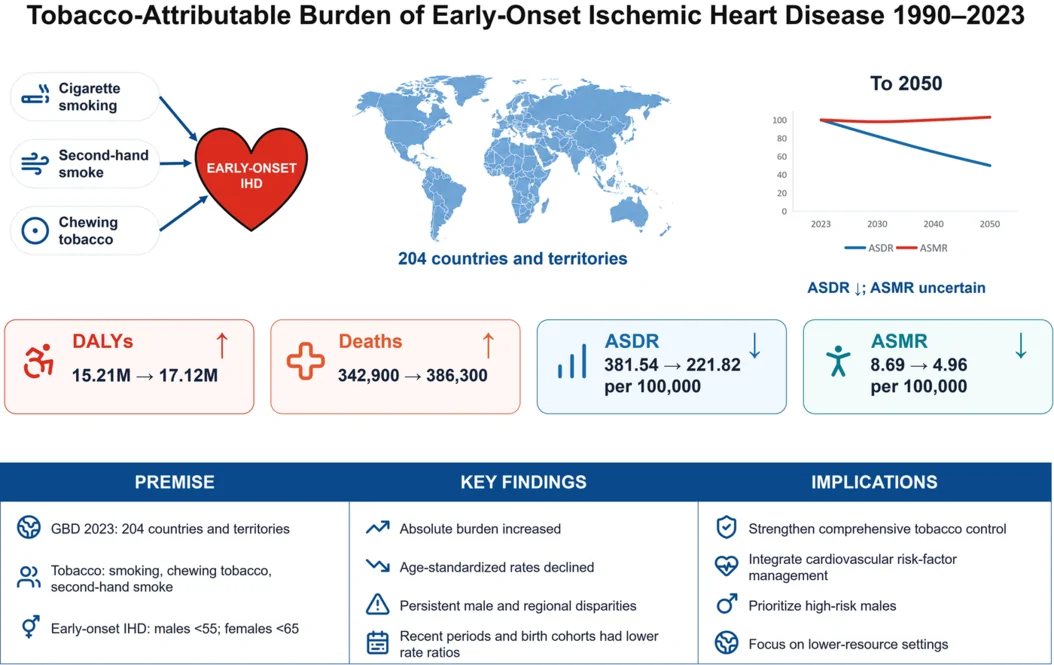

## 1 Introduction

Ischemic heart disease (IHD) remains the leading cause of global mortality and disability [1]. A particularly concerning trend is the rising incidence of early‐onset IHD, defined as onset before age 55 in men and 65 in women, especially among younger populations [2, 3]. This demographic shift results in substantial societal and economic costs due to premature death and the loss of productive life years [4].

Tobacco exposure is among the most important preventable risk factors for IHD [5]. In the GBD risk hierarchy, tobacco includes smoking, chewing tobacco, and secondhand smoke. Comprehensive tobacco‐control policies have reduced exposure in many high‐SDI settings [6], but the burden among young males remains of particular concern in several low‐ and middle‐SDI settings [7, 8].

The health burden imposed by tobacco exhibits complex spatial and temporal variations, which must be understood to guide effective interventions. Previous research has documented the overall IHD burden and the cardiovascular risks of smoking [9], but studies focused on early‐onset IHD attributable to the broader GBD tobacco category remain scarce. Important evidence gaps persist regarding long‐term trends, regional differences, and the distinct influences of age, period, and birth cohort.

To address these gaps, we used GBD 2023 data to examine tobacco‐attributable early‐onset IHD across 204 countries and territories from 1990 to 2023. We evaluated temporal trends, applied age‐period‐cohort (APC) modeling to describe age, period, and cohort patterns, conducted a common‐age‐range sensitivity analysis, and projected age‐standardized burden through 2050.

## 2 Materials and Methods

### 2.1 Study Design and Data Source

This secondary analysis used publicly available estimates from the Global Burden of Disease Study 2023 (GBD 2023), which quantified the burden of 375 diseases and injuries attributable to 88 modifiable risk factors across 204 countries and territories from 1990 to 2023. The core GBD 2023 report describes the methodological framework for cause‐of‐death estimation, non‐fatal burden modeling, uncertainty propagation, and comparative risk assessment [10, 11, 12]. GBD data are anonymized and publicly accessible through the GBD Results Tool (https://ghdx.healthdata.org/gbd-results-tool); therefore, additional informed consent was not required.

### 2.2 Case Definition, Exposure, and Target Population

The primary outcome was early‐onset IHD, including acute myocardial infarction, chronic stable angina, and IHD‐related heart failure. Early‐onset IHD was restricted to individuals younger than 55 years in males and younger than 65 years in females, consistent with the prespecified study definition. The exposure was the GBD level‐2 risk category tobacco, which comprises smoking, chewing tobacco, and second‐hand smoke. Emerging products such as electronic cigarettes are not modeled as a separate exposure in GBD 2023 and therefore could not be incorporated into the attributable‐burden estimates or projections.

Because the primary sex‐specific definitions use different age windows, direct male‐female comparisons were interpreted cautiously. As a sensitivity analysis, ASDR, ASMR, EAPC, and male‐to‐female rate ratios were recalculated using the common age range of 40–54 years for both sexes.

### 2.3 Metrics and Classifications

We assessed tobacco‐attributable deaths and disability‐adjusted life years (DALYs) in absolute numbers and as age‐standardized rates per 100 000 population using the GBD standard population. ASMR denotes the age‐standardized mortality rate, and ASDR denotes the age‐standardized DALY rate. Estimates were summarized globally, for 21 GBD regions, across 204 countries and territories, and by the five SDI strata.

SDI, a composite indicator based on income per capita, educational attainment, and fertility, was used to categorize locations into high (SDI > 0.81), high‐middle (0.70−0.81), middle (0.61−0.70), low‐middle (0.46−0.60), and low (< 0.46) SDI groups.

### 2.4 Statistical Analysis

We summarized estimates with 95% uncertainty intervals (UIs) derived from 250 posterior draws. Changes from 1990 to 2023 were assessed using absolute and percentage changes. EAPC was estimated from the log‐linear model ln(ASR) = *α* + *β* × year + *ε*, with EAPC = 100 × [exp(*β*) − 1]. An EAPC was considered statistically significant when its 95% CI excluded zero. Joinpoint regression was used to identify temporal changes and to estimate AAPCs. LOESS smoothing and Spearman's correlation were used to examine the relationship between age‐standardized burden and SDI.

Sex‐specific APC models were fitted to age‐specific event counts and population denominators. Because age, period, and cohort are linearly dependent, we report estimable functions rather than mutually adjusted coefficients: age deviations, period and cohort deviations, period and cohort rate ratios, net drift (the overall annual percentage change), and local drifts (age‐specific annual percentage changes), each with 95% CIs. Wald tests evaluated whether net drift equaled zero, whether age, period, and cohort deviations equaled zero, whether period and cohort rate ratios equaled one, and whether local drifts differed from net drift.

For projections from 2024 through 2050, Bayesian APC models with second‐order random‐walk smoothing priors for age, period, and cohort effects were fitted using integrated nested Laplace approximations. Posterior point estimates and uncertainty bands were generated for sex‐specific ASDRs and ASMRs.

Analyses were conducted in R version 4.3.2 using packages including ggplot2, Epi, Joinpoint, BAPC, and INLA. Statistical tests were two‐sided, and *p* < 0.05 was considered statistically significant.

## 3 Results

### 3.1 Global Burden of Early‐Onset IHD Attributable to Tobacco

Between 1990 and 2023, the global absolute burden of tobacco‐attributable early‐onset IHD increased, while age‐standardized rates declined. DALYs increased by 12.6%, from 15.21 million (95% UI 12.58−18.27 million) to 17.12 million (95% UI 13.89−20.82 million). Deaths increased by 12.7%, from 342 900 (95% UI 282 700−414 000) to 386 300 (95% UI 313 600−471 500).

**Figure 1:**
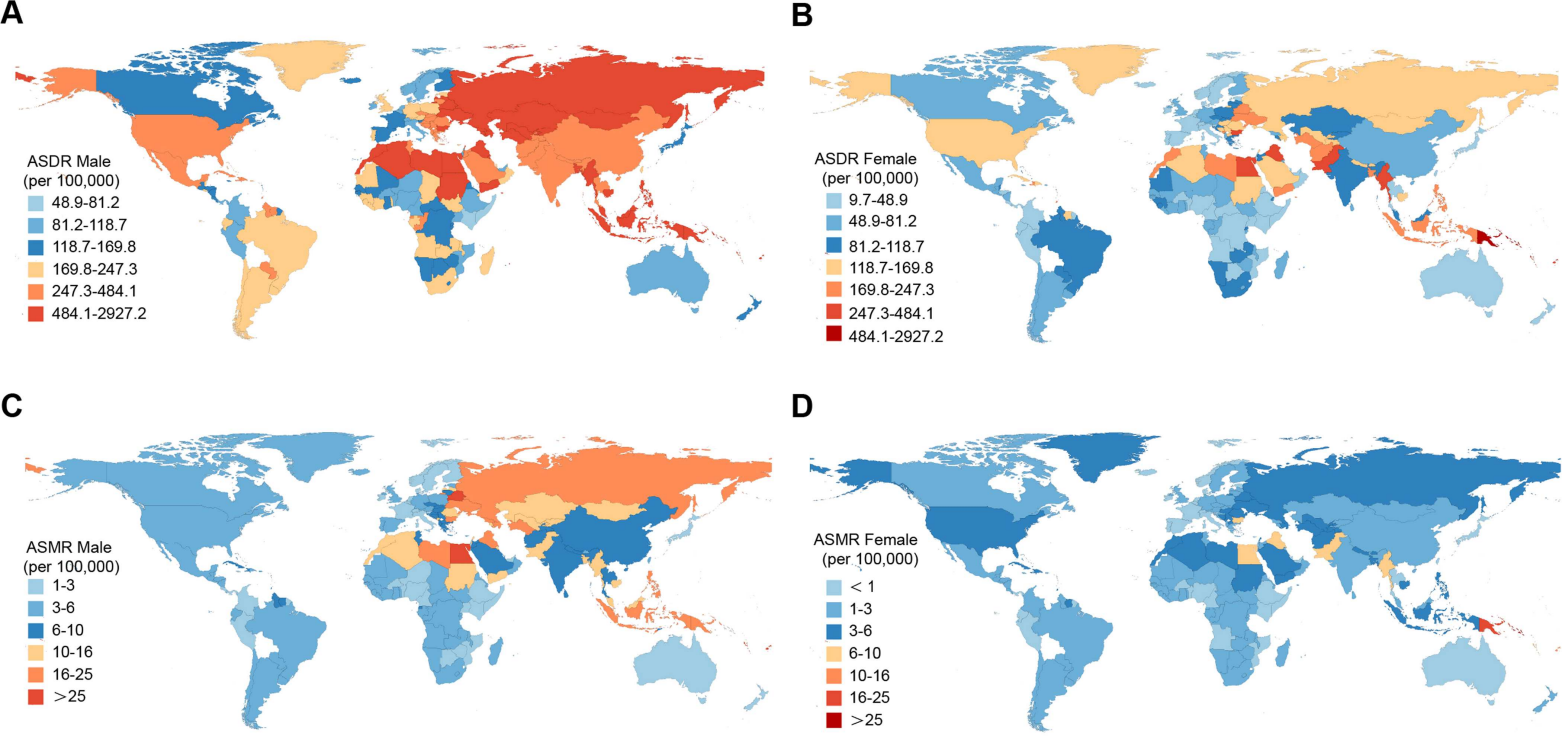
National distribution of the tobacco‐attributable early‐onset IHD burden in 2023. Panels show ASDR and ASMR from 1990 to 2023. (A) Male ASDR. (B) Female ASDR. (C) Male ASMR. (D) Female ASMR.

The global ASDR fell from 381.54 per 100 000 (95% UI 316.25−457.63) in 1990 to 221.82 (95% UI 179.73−270.05) in 2023, with an EAPC of −1.84% (95% CI −1.94 to −1.73). The ASMR declined from 8.69 per 100 000 (95% UI 7.18−10.48) to 4.96 (95% UI 4.03−6.07), with an EAPC of −1.88% (95% CI −1.98 to −1.77). National distributions are shown in Figure 1 and detailed global and regional estimates are provided in Tables 1 and 2.

**Table 1:** Global and regional burden of tobacco‐attributable DALYs from early‐onset IHD from 1990 to 2023.

Location	Number, 1990, 10^4 (95% UI)	Number, 2023, 10^4 (95% UI)	ASRs, 1990, per 100 000 (95% UI)	ASRs, 2023, per 100 000 (95% UI)	EAPC, 1990−2023 (95% CI)
Global	1520.73 (1258.05, 1826.80)	1712.48 (1388.53, 2082.34)	381.54 (316.25, 457.63)	221.82 (179.73, 270.05)	−1.84 (−1.94 to −1.73)
High SDI	785.59 (681.39, 900.18)	562.82 (479.47, 653.46)	422.47 (366.89, 483.42)	192.28 (163.77, 223.52)	−2.85 (−3.06 to −2.65)
Low‐middle SDI	179.93 (126.51, 248.77)	237.54 (166.75, 327.72)	388.00 (273.54, 535.17)	238.88 (167.79, 329.10)	−1.27 (−1.51 to −1.03)
High‐middle SDI	274.89 (210.07, 351.97)	310.19 (243.31, 385.63)	332.34 (254.88, 425.06)	178.43 (140.02, 222.16)	−1.97 (−2.04 to −1.9)
Middle SDI	151.27 (114.91, 194.72)	343.45 (266.15, 433.57)	386.74 (294.64, 496.74)	367.53 (284.61, 464.25)	−0.03 (−0.12 to 0.07)
Low SDI	126.6 (82.54, 180.91)	256.7 (175.78, 363.40)	285.22 (187.38, 406.23)	231.52 (159.23, 326.84)	−0.54 (−0.67 to −0.42)
Female	405.8 (324.96, 507.98)	405.3 (318.44, 501.43)	200.83 (161.10, 250.93)	97.98 (76.81, 121.46)	−2.38 (−2.46 to −2.29)
Male	1114.8 (933.10, 1318.82)	1307.0 (1070.10, 1580.90)	579.70 (486.38, 684.29)	357.62 (292.59, 432.99)	−1.66 (−1.78 to −1.53)
East Asia	268.23 (171.56, 388.78)	324.46 (231.06, 436.78)	274.44 (176.18, 396.94)	184.13 (131.16, 247.97)	−1.37 (−1.46 to −1.29)
Southeast Asia	108.25 (71.93, 153.93)	268.25 (195.06, 358.60)	356.30 (238.05, 504.80)	374.02 (271.91, 500.02)	0.41 (0.32 to 0.49)
Oceania	2.86 (1.79, 4.31)	8.92 (5.68, 13.43)	727.29 (457.34, 1097.88)	825.37 (525.77, 1240.89)	0.3 (0.22 to 0.37)
Caribbean	10.30 (7.99, 12.78)	10.18 (7.48, 13.69)	418.83 (325.16, 519.08)	218.57 (160.89, 294.01)	−2.17 (−2.44 to −1.9)
Central Asia	35.22 (29.22, 41.55)	34.46 (28.59, 40.61)	754.31 (627.48, 887.17)	381.75 (317.22, 449.11)	−2.75 (−3.21 to −2.29)
Eastern Europe	179.65 (153.90, 207.73)	119.75 (100.50, 139.40)	803.73 (691.18, 924.73)	497.83 (419.23, 576.94)	−2.56 (−3.23 to −1.88)
Western Europe	128.25 (113.03, 142.83)	49.55 (41.96, 57.74)	326.65 (288.05, 363.52)	99.47 (84.41, 115.92)	−3.94 (−4.1 to −3.77)
Central Europe	96.08 (83.90, 108.15)	32.27 (27.70, 36.85)	781.02 (684.76, 875.29)	231.50 (198.83, 264.64)	−4.05 (−4.21 to −3.9)
Australasia	5.89 (5.09, 6.67)	2.64 (2.03, 3.30)	315.86 (273.08, 357.52)	78.74 (60.96, 98.18)	−4.26 (−4.39 to −4.12)
High‐income Asia Pacific	26.17 (22.37, 30.37)	14.81 (11.79, 18.08)	145.57 (124.27, 169.08)	68.01 (54.09, 83.16)	−2.58 (−2.75 to −2.4)
Southern Latin America	13.54 (11.00, 16.17)	8.94 (7.07, 11.18)	336.52 (274.13, 400.51)	127.84 (101.50, 159.41)	−2.95 (−3.11 to −2.79)
High‐income North America	115.70 (100.66, 129.42)	71.82 (55.25, 90.57)	457.47 (398.50, 510.84)	181.96 (140.53, 228.75)	−2.97 (−3.09 to −2.85)
Andean Latin America	2.86 (2.09, 3.87)	4.34 (3.18, 5.78)	122.63 (89.46, 165.68)	72.13 (52.90, 96.09)	−2.16 (−2.58 to −1.74)
Central Latin America	24.22 (19.59, 29.25)	33.90 (26.79, 41.70)	254.07 (205.51, 306.89)	139.07 (110.07, 170.76)	−1.89 (−2.15 to −1.62)
North Africa and Middle East	126.24 (93.97, 166.10)	221.69 (172.83, 282.90)	630.87 (470.65, 827.86)	363.65 (282.94, 464.77)	−1.82 (−1.93 to −1.72)
Tropical Latin America	49.89 (41.73, 58.59)	35.25 (27.02, 44.91)	485.82 (406.73, 570.23)	151.33 (116.23, 192.43)	−3.81 (−3.94 to −3.69)
Eastern Sub‐Saharan Africa	7.90 (4.80, 12.22)	21.94 (13.63, 34.41)	82.54 (50.47, 127.22)	85.01 (52.78, 133.31)	0.24 (0.11 to 0.36)
Western Sub‐Saharan Africa	11.44 (6.53, 18.49)	24.12 (14.04, 40.29)	109.26 (62.57, 177.14)	84.64 (49.47, 141.34)	−0.88 (−1.07 to −0.69)
Southern Sub‐Saharan Africa	6.90 (4.93, 9.42)	10.67 (7.72, 14.26)	231.46 (166.14, 315.29)	143.99 (104.34, 191.98)	−1.58 (−1.81 to −1.35)
South Asia	297.92 (200.23, 419.03)	404.96 (270.83, 577.67)	414.42 (279.30, 582.33)	245.98 (164.65, 350.43)	−1.41 (−1.7 to −1.12)
Central Sub‐Saharan Africa	3.21 (1.57, 5.48)	9.53 (4.47, 17.03)	111.31 (54.88, 188.36)	110.30 (52.34, 196.01)	0.1 (−0.1 to 0.31)

**Table 2:** Global and regional burden of tobacco‐attributable deaths from early‐onset IHD from 1990 to 2023.

Location	Number, 1990, 10^4 (95% UI)	Number, 2023, 10^4 (95% UI)	ASRs, 1990, per 100 000 (95% UI)	ASRs, 2023, per 100 000 (95% UI)	EAPC, 1990−2023 (95% CI)
Global	34.29 (28.27, 41.40)	38.63 (31.36, 47.15)	8.69 (7.18, 10.48)	4.96 (4.03, 6.07)	−1.88 (−1.98 to −1.77)
High SDI	17.81 (15.38, 20.42)	12.57 (10.72, 14.52)	9.55 (8.26, 10.94)	4.20 (3.58, 4.86)	−2.95 (−3.15 to −2.74)
Low‐middle SDI	4.07 (2.84, 5.65)	5.47 (3.81, 7.56)	8.98 (6.28, 12.45)	5.55 (3.87, 7.67)	−1.24 (−1.47 to −1)
High‐middle SDI	6.11 (4.62, 7.88)	6.89 (5.34, 8.57)	7.57 (5.73, 9.75)	3.91 (3.03, 4.88)	−2.06 (−2.13 to −1.98)
Middle SDI	3.36 (2.54, 4.36)	7.76 (6.00, 9.82)	8.77 (6.66, 11.35)	8.29 (6.41, 10.50)	−0.04 (−0.14 to 0.07)
Low SDI	2.89 (1.88, 4.13)	5.90 (4.03, 8.39)	6.68 (4.38, 9.54)	5.46 (3.74, 7.74)	−0.51 (−0.64 to −0.38)
Female	10.38 (8.29, 13.07)	10.35 (8.21, 12.86)	5.17 (4.13, 6.50)	2.47 (1.96, 3.08)	−2.42 (−2.5 to −2.34)
Male	23.91 (19.98, 28.33)	28.28 (23.15, 34.29)	12.56 (10.52, 14.85)	7.70 (6.30, 9.34)	−1.66 (−1.79 to −1.54)
East Asia	5.88 (3.68, 8.64)	7.07(4.89, 9.71)	6.15 (3.87, 9.02)	3.90 (2.70, 5.36)	−1.5 (−1.6 to −1.4)
Southeast Asia	2.43 (1.60, 3.48)	6.13 (4.44, 8.24)	8.17 (5.42, 11.66)	8.51 (6.15, 11.43)	0.38 (0.29 to 0.46)
Oceania	0.07 (0.04, 0.10)	0.21 (0.13, 0.31)	17.28 (10.77, 26.32)	19.53 (12.38, 29.37)	0.26 (0.18 to 0.34)
Caribbean	0.23 (0.18, 0.29)	0.23 (0.16, 0.30)	9.43 (7.25, 11.78)	4.76 (3.49, 6.43)	−2.27 (−2.55 to −1.99)
Central Asia	0.76 (0.63, 0.91)	0.73 (0.61, 0.87)	16.23 (13.45, 19.27)	8.12 (6.74, 9.60)	−2.83 (−3.31 to −2.36)
Eastern Europe	4.11 (3.52, 4.79)	2.74 (2.29, 3.20)	18.02 (15.48, 20.83)	11.22 (9.40, 13.02)	−2.55 (−3.24 to −1.86)
Western Europe	2.92 (2.59, 3.25)	1.08 (0.92, 1.25)	7.32 (6.48, 8.11)	2.10 (1.81, 2.44)	−4.09 (−4.28 to −3.91)
Central Europe	2.22 (1.94, 2.52)	0.75 (0.65, 0.86)	17.88 (15.63, 20.10)	5.30 (4.57, 6.07)	−4.04 (−4.19 to −3.89)
Australasia	0.14 (0.12, 0.16)	0.06 (0.04, 0.07)	7.39 (6.45, 8.37)	1.71 (1.32, 2.13)	−4.46 (−4.64 to −4.29)
High‐income Asia Pacific	0.55 (0.47, 0.63)	0.30 (0.24, 0.36)	3.04 (2.59, 3.50)	1.34 (1.08, 1.62)	−2.72 (−2.91 to −2.53)
Southern Latin America	0.31 (0.25, 0.37)	0.20 (0.16, 0.25)	7.72 (6.25, 9.24)	2.80 (2.22, 3.50)	−3.06 (−3.22 to −2.9)
High‐income North America	2.66 (2.32, 2.99)	1.68 (1.29, 2.12)	10.56 (9.22, 11.84)	4.13 (3.18, 5.19)	−3.07 (−3.21 to −2.92)
Andean Latin America	0.06 (0.04, 0.08)	0.09 (0.07, 0.12)	2.69 (1.95, 3.65)	1.51 (1.11, 2.03)	−2.3 (−2.73 to −1.86)
Central Latin America	0.54 (0.44, 0.66)	0.76 (0.60, 0.94)	5.88 (4.74, 7.14)	3.09 (2.45, 3.81)	−2.04 (−2.33 to −1.75)
North Africa and Middle East	2.84 (2.11, 3.77)	4.88 (3.79, 6.26)	14.55 (10.80, 19.28)	8.13 (6.31, 10.47)	−1.91 (−2.02 to −1.8)
Tropical Latin America	1.12 (0.94, 1.32)	0.84 (0.64, 1.07)	11.16 (9.34, 13.15)	3.55 (2.73, 4.53)	−3.74 (−3.86 to −3.62)
Eastern Sub‐Saharan Africa	0.17 (0.10, 0.27)	0.48 (0.29, 0.76)	1.84 (1.11, 2.87)	1.93 (1.18, 3.05)	0.29 (0.17 to 0.42)
Western Sub‐Saharan Africa	0.26 (0.15, 0.42)	0.54 (0.31, 0.91)	2.54 (1.43, 4.15)	1.95 (1.13, 3.29)	−0.91 (−1.11 to −0.71)
Southern Sub‐Saharan Africa	0.15 (0.11, 0.21)	0.23 (0.16, 0.31)	5.17 (3.66, 7.18)	3.12 (2.21, 4.21)	−1.68 (−1.93 to −1.42)
South Asia	6.79 (4.53, 9.59)	9.43 (6.28, 13.47)	9.70 (6.49, 13.71)	5.80 (3.86, 8.28)	−1.37 (−1.65 to −1.08)
Central Sub‐Saharan Africa	0.07 (0.03, 0.12)	0.21 (0.10, 0.37)	2.45 (1.19, 4.18)	2.45 (1.14, 4.38)	0.12 (−0.09 to 0.32)

### 3.2 Burden by SDI Level and Region

**Figure 2:**
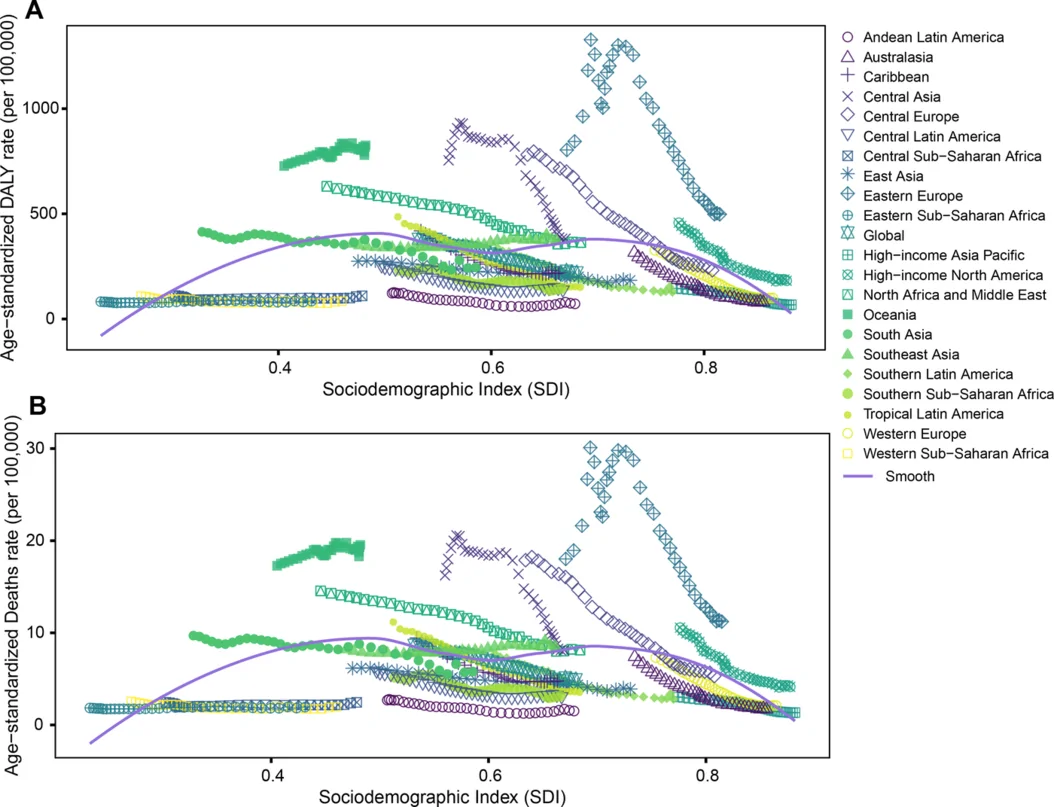
Association between SDI and the tobacco‐attributable burden of early‐onset IHD across 21 GBD regions, 1990−2023. (A) ASDR. (B) ASMR. Curves were fitted using LOESS smoothing.

The association between SDI and tobacco‐attributable early‐onset IHD burden was non‐linear, with the fitted curves rising across lower SDI values and declining at higher SDI values (Figure 2). In 2023, the highest regional ASDRs were observed in Oceania (825.37 per 100 000), Eastern Europe (497.83), Central Asia (381.75), Southeast Asia (374.02), and North Africa and the Middle East (363.65). The lowest ASDRs were observed in high‐income Asia Pacific (68.01), Andean Latin America (72.13), Australasia (78.74), Western sub‐Saharan Africa (84.64), and Eastern sub‐Saharan Africa (85.01). ASMR patterns were similar.

**Figure 3:**
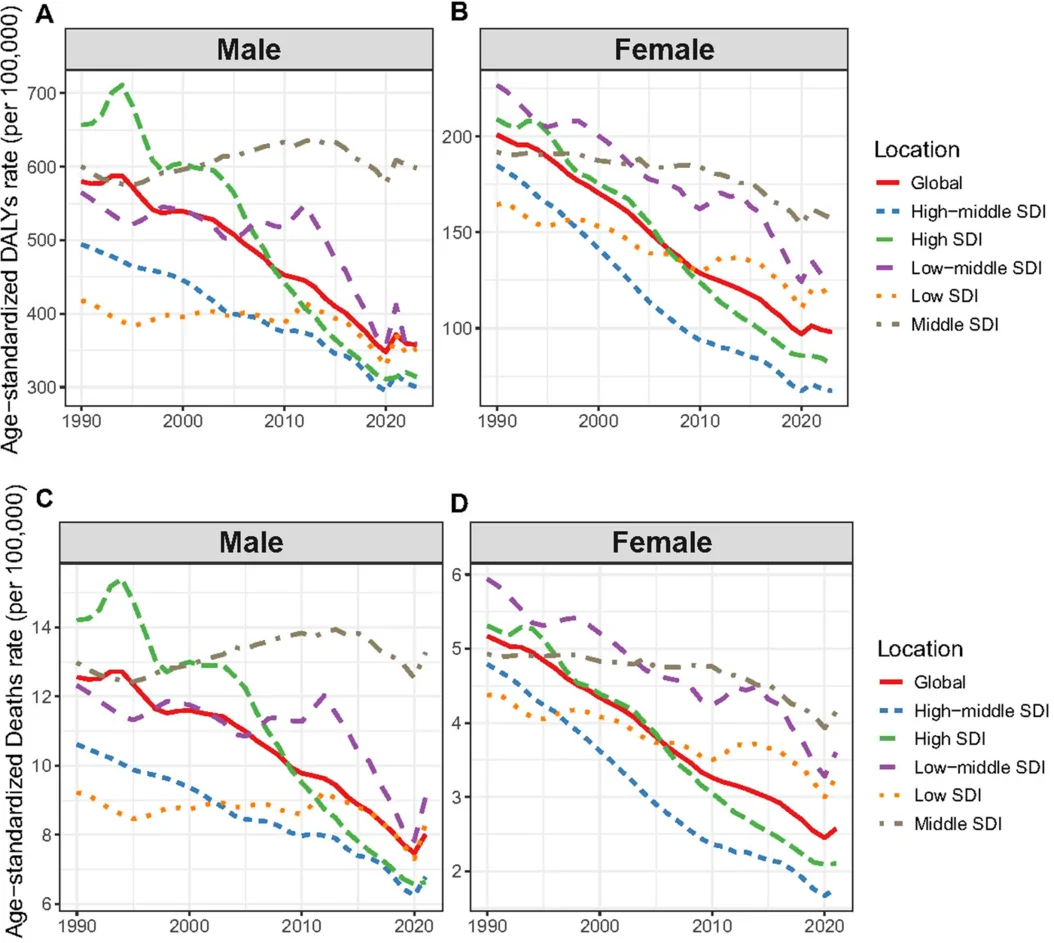
Temporal trends in tobacco‐attributable early‐onset IHD burden by sex and SDI level, 1990−2023. (A) Male ASDR. (B) Female ASDR. (C) Male ASMR. (D) Female ASMR.

High‐SDI settings achieved the largest sustained reductions: ASDR decreased from 422.47 to 192.28 per 100 000 (EAPC −2.85%, 95% CI −3.06 to −2.65) and ASMR decreased from 9.55 to 4.20 (EAPC −2.95%, 95% CI −3.15 to −2.74). Reductions were smaller in low‐middle‐SDI (ASDR EAPC −1.27%), low‐SDI (−0.54%), and especially middle‐SDI settings (−0.03%, 95% CI −0.12 to 0.07), where the long‐term change in ASDR was not statistically significant (Tables 1 and 2 and Figures 2 and 3).

### 3.3 Sex Disparities and Sensitivity Analysis

Using the prespecified sex‐specific definitions, male ASDR declined from 579.70 to 357.62 per 100 000 (EAPC −1.66%, 95% CI −1.78 to −1.53), whereas female ASDR declined from 200.83 to 97.98 (EAPC −2.38%, 95% CI −2.46 to −2.29). Male ASMR decreased from 12.56 to 7.70 per 100 000 (EAPC −1.66%, 95% CI −1.79 to −1.54), and female ASMR decreased from 5.17 to 2.47 (EAPC −2.42%, 95% CI −2.50 to −2.34). The male‐to‐female ratios increased from 2.89 to 3.65 for ASDR and from 2.43 to 3.12 for ASMR, although these primary comparisons use different age windows and should not be interpreted as like‐for‐like estimates.

In the sensitivity analysis restricted to ages 40−54 years in both sexes, male ASDR declined from 2303.76 to 1405.90 per 100 000, while female ASDR declined from 427.39 to 216.73. The male‐to‐female ASDR ratio increased from 5.39 to 6.49. Corresponding ASMRs declined from 52.59 to 31.97 in males and from 9.49 to 4.81 in females, with the male‐to‐female ratio increasing from 5.54 to 6.65. Thus, the higher male burden and widening relative disparity persisted when the age range was harmonized (Table S1).

### 3.4 Temporal Trends and Regional Heterogeneity

**Figure 4:**
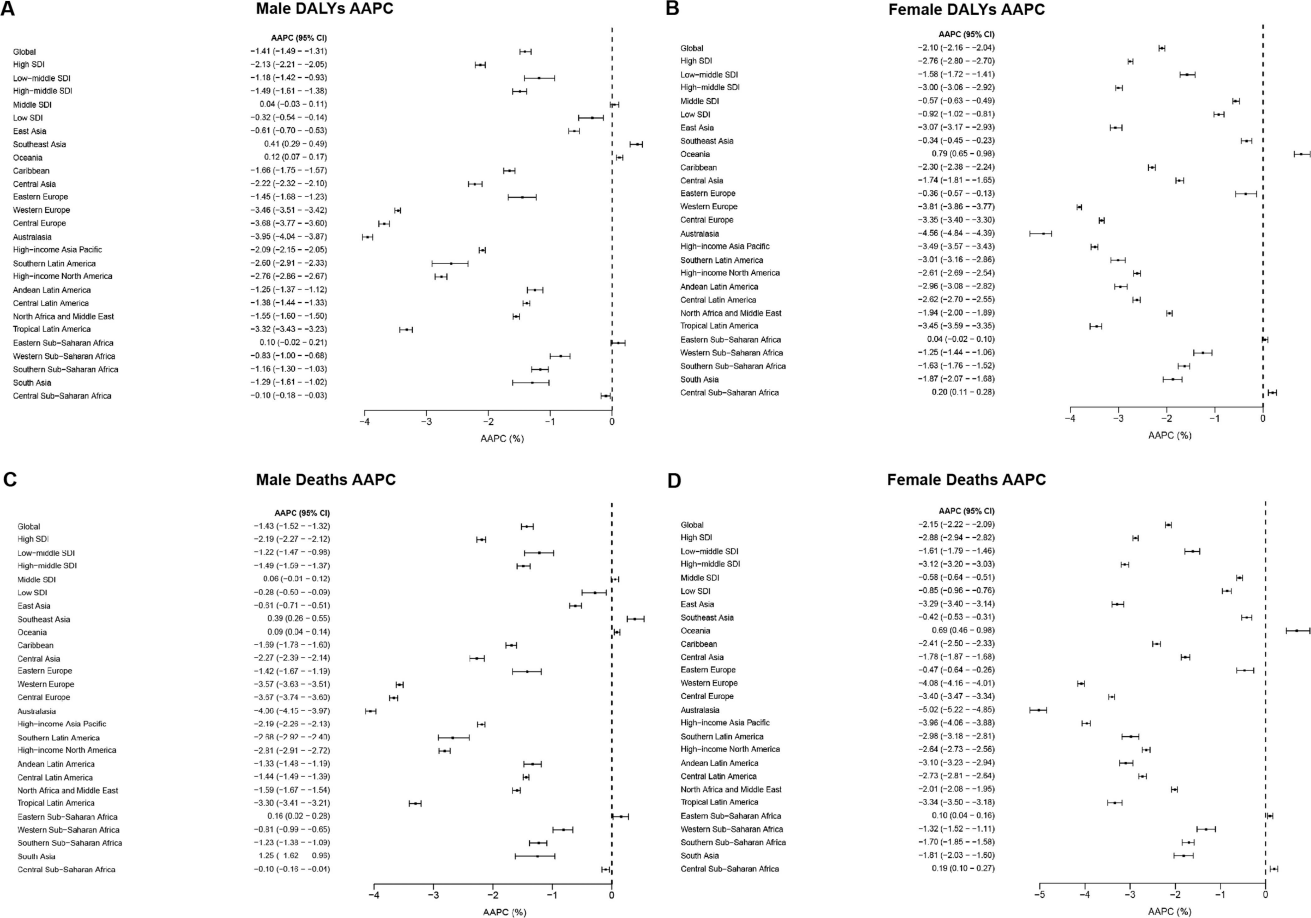
AAPCs in tobacco‐attributable early‐onset IHD burden across global, SDI, and regional groups from 1990 to 2023. (A) Male ASDR. (B) Female ASDR. (C) Male ASMR. (D) Female ASMR. Error bars indicate 95% CIs.

From 1990 to 2023, global AAPCs were −1.41% (95% CI −1.49 to −1.31) for male ASDR, −2.10% (−2.16 to −2.04) for female ASDR, −1.43% (−1.52 to −1.32) for male ASMR, and −2.15% (−2.22 to −2.09) for female ASMR (Figure 4). Australasia, Central Europe, and Western Europe showed some of the steepest declines, whereas male trends increased in Southeast Asia and Oceania and changed little in several low‐SDI regions. The temporal trajectories by sex and SDI are shown in Figure 3.

### 3.5 Decomposition Analysis: Demographic Versus Epidemiological Drivers

**Figure 5:**
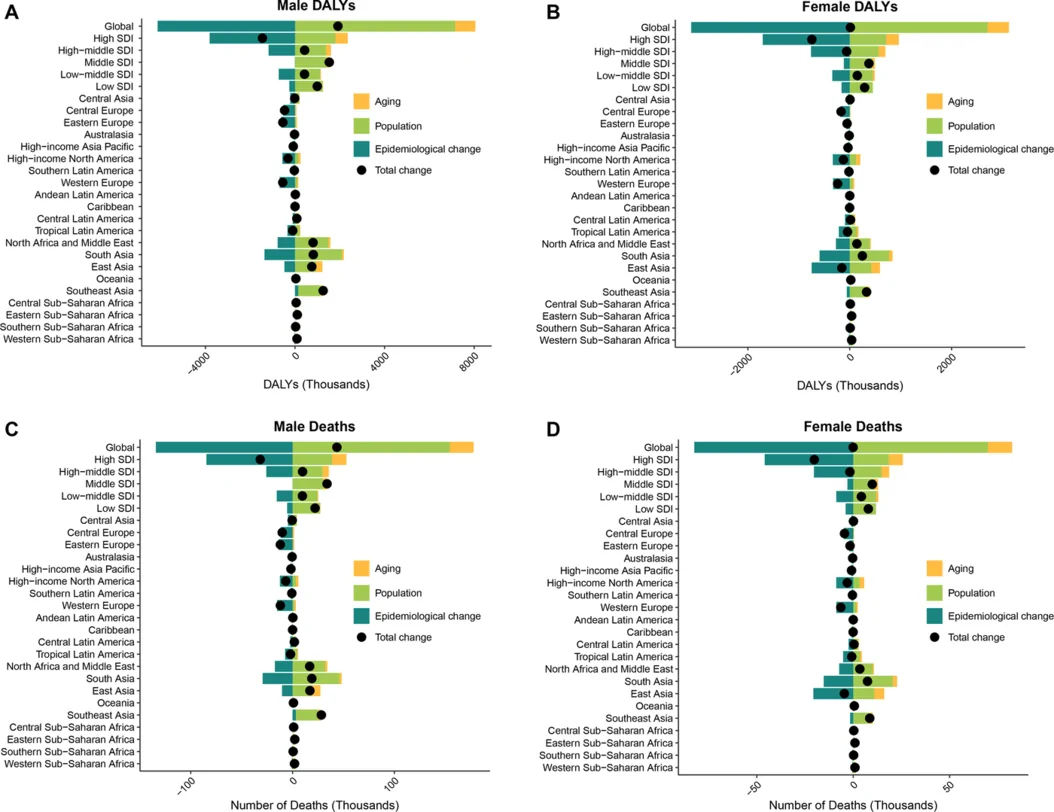
Decomposition of changes in tobacco‐attributable DALYs and deaths, 1990−2023. (A) Male DALYs. (B) Female DALYs. (C) Male deaths. (D) Female deaths.

Decomposition analyses from 1990 to 2023 indicated that population growth and aging were the primary drivers increasing absolute DALYs and deaths, while favorable epidemiological changes (declines in age‐specific rates) exerted a negative, partially offsetting effect. For men (Figure 5A,C), the net global increase in absolute burden resulted from strong positive contributions from population growth and aging, which were partly counterbalanced by rate reductions. In high‐SDI regions, the negative epidemiological component exceeded demographic pressures, leading to net decreases—a pattern also seen in Western Europe, high‐income Asia Pacific, Australasia, and high‐income North America. In South Asia and low‐middle SDI settings, the positive contributions from population growth (and, to a lesser extent, aging) outweighed the effect of rate reductions, resulting in net increases. Across sub‐Saharan Africa, population growth was the dominant positive driver, with epidemiological changes showing minimal or modestly negative contributions. For women (Figure 5B,D), the patterns were similar, with population growth and aging contributing positively in most regions and rate reductions contributing negatively; the contribution of aging was more prominent in several regions. Western Europe and high‐income Asia Pacific showed clear net decreases where negative epidemiological contributions surpassed demographic increases, while sub‐Saharan Africa and South Asia exhibited net increases driven mainly by population growth.

### 3.6 Age‐Period‐Cohort Analysis

The APC models identified significant, favorable overall temporal trends. Net drifts for DALYs were −1.524% per year (95% CI −1.632 to −1.417) in males and −2.291% per year (95% CI −2.340 to −2.241) in females (Supporting Information S1: Figures S1−S2). Net drifts for deaths were −1.541% per year (95% CI −1.660 to −1.423) in males and −2.299% per year (95% CI −2.355 to −2.242) in females (Supporting Information S1: Figures S3−S4).

Period rate ratios declined progressively after the early‐2000s reference period in both sexes, with a steeper reduction among females. Cohort rate ratios also decreased across successive birth cohorts: they fell below one after approximately the mid‐1960s in males and after approximately 1960 in females, and remained lower in later cohorts. Age deviations were positive in early‐to‐middle adulthood and declined at older ages within each sex‐specific early‐onset window. Wald tests supported non‐zero net drift and significant age, period, and cohort components (all *p* < 0.001). Local drifts also varied by age, indicating that the pace of decline was not uniform across age groups.

### 3.7 Projected Burden to 2050

**Figure 6:**
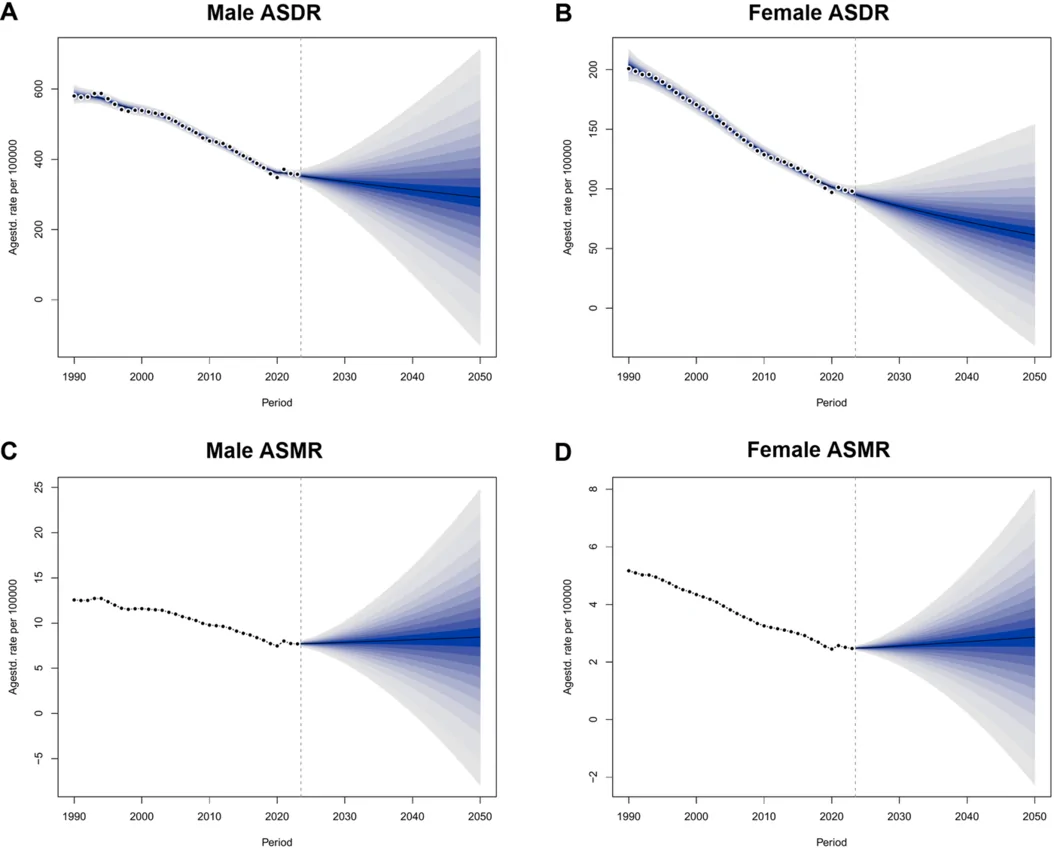
Observed and projected age‐standardized rates of tobacco‐attributable early‐onset IHD, 1990−2050. (A) Male ASDR. (B) Female ASDR. (C) Male ASMR. (D) Female ASMR. Solid lines show Bayesian APC posterior point estimates, and shaded bands show predictive uncertainty.

Bayesian APC projections suggested that ASDRs would continue to decline in both sexes through 2050. In contrast, ASMR point estimates were broadly stable or increased slightly after 2023. UIs widened substantially with the projection horizon, so the long‐term mortality pattern should be interpreted cautiously (Figure 6).

## 4 Discussion

This analysis of GBD 2023 data shows a persistent divergence in the global burden of tobacco‐attributable early‐onset IHD: ASDR and ASMR declined substantially from 1990 to 2023, but absolute DALYs and deaths increased by approximately 13%. The updated estimates also demonstrate pronounced heterogeneity by SDI and region, a consistently higher burden in males, significant age‐period‐cohort structure, and different projected trajectories for standardized DALY and mortality rates.

The largest improvements occurred in high‐SDI settings. This progress is consistent with comprehensive tobacco‐control strategies, including national policy initiatives, the WHO Framework Convention on Tobacco Control, and the MPOWER package [13, 14, 15]. Tobacco taxation is among the most effective and cost‐effective measures for reducing consumption and preventing tobacco‐attributable deaths [16, 17]. Greater access to statins, antihypertensive and antiplatelet therapies, and timely reperfusion has also contributed to declining coronary mortality [18, 19]. Comprehensive smoke‐free legislation is associated with fewer acute coronary events and cardiovascular deaths [20]. The decline in period rate ratios after the early 2000s is temporally compatible with these policy and treatment gains, although the ecological APC design cannot establish causality.

Progress was much slower in middle‐, low‐middle‐, and low‐SDI settings, and the near‐zero long‐term EAPC in middle‐SDI settings is especially concerning. Tobacco‐control implementation remains uneven across world regions [21], reflecting differences in socioeconomic development, governance capacity, and public‐health prioritization [22]. Persistently high smoking prevalence among men and limited access to essential cardiovascular medicines and acute care further impede progress [23, 24]. Lower availability and affordability of key cardiovascular medicines are associated with worse cardiovascular outcomes [25]. A dual strategy of strengthening fiscal and legislative tobacco control while ensuring reliable access to essential therapies is therefore critical [16].

The higher male burden is robust to the common‐age‐range sensitivity analysis, reducing concern that it is solely an artifact of the different primary age windows. The pattern is consistent with historically higher tobacco use and cumulative exposure among males [26, 27]. Women may nevertheless have greater relative coronary risk at a given level of smoking exposure [28], underscoring the importance of prevention in both sexes. Absolute comparisons under the primary definition remain inherently asymmetric because it includes males younger than 55 years and females younger than 65 years.

The progressively lower period and cohort rate ratios indicate that recent calendar periods and later‐born generations experienced a lower tobacco‐attributable burden than the reference groups. These patterns likely reflect reduced combustible‐tobacco exposure, smoke‐free policies, improved cardiovascular prevention, and changes in competing risks. Second‐hand smoke remains an important contributor to cardiovascular burden [29, 30, 31], while the cardiovascular effects of electronic nicotine delivery systems remain an active area of research [32]. Potential risks and dual use with conventional cigarettes warrant continued surveillance, especially among young people [33, 34].

### 4.1 Clinical and Public Health Implications

The findings support a dual population‐level and clinical strategy. At the population level, tobacco‐control policies should further strengthen taxation, comprehensive smoke‐free legislation and enforcement, restrictions on tobacco marketing and promotion, and protection from second‐hand smoke exposure. These measures may be particularly important in countries and regions where age‐standardized rates have plateaued or where declines have slowed, as continued exposure and uneven implementation of tobacco‐control policies may limit further reductions in disease burden. At the clinical level, healthcare systems should routinely identify tobacco exposure at an early stage, provide accessible and sustained cessation support, and integrate cessation treatment with active management of major cardiovascular risk factors, including dyslipidemia, hypertension, and impaired glucose metabolism. Particular attention may be warranted for high‐risk male populations and individuals living in lower‐resource settings, where access to preventive services may remain limited. Although the projected decline in ASDR is encouraging and suggests a continued reduction in nonfatal health loss, the broadly stable or slightly increasing ASMR point estimates indicate that improvements in disability burden may not necessarily translate into equivalent reductions in premature mortality. Moreover, the widening prediction intervals with longer projection horizons highlight substantial uncertainty. Therefore, sustained tobacco control, early cardiovascular risk assessment, and integrated primary prevention will remain necessary to achieve further and more equitable reductions in mortality.

### 4.2 Strengths and Limitations

This study combines updated GBD 2023 estimates with global, regional, sex‐specific, APC, sensitivity, and projection analyses. Several limitations should be considered. First, GBD estimates depend on the quality and availability of source data and statistical modeling, especially in data‐sparse settings. Second, the primary early‐onset definitions use different age windows for males and females; although the 40−54‐year sensitivity analysis confirmed the direction of the sex disparity, comparisons based on the primary definition remain asymmetric. Third, APC components describe population‐level temporal patterns and should not be interpreted as causal effects. Fourth, electronic cigarettes and other emerging products were not modeled separately. Finally, BAPC uncertainty widened markedly toward 2050, limiting the precision of long‐range projections.

## 5 Conclusions

From 1990 to 2023, age‐standardized rates of tobacco‐attributable early‐onset IHD declined, but absolute DALYs and deaths increased. More favorable period and cohort patterns were evident in recent years and later‐born generations, yet progress was uneven, and the male burden remained substantially higher even within a common age range. Strengthening comprehensive tobacco control and integrating cessation with lipid, blood‐pressure, and glucose management should be prioritized in high‐burden and lower‐resource settings. Continued surveillance is essential because long‐term mortality projections remain uncertain and emerging tobacco products are not fully captured in current GBD estimates.

## Author Contributions

Methodology: Xin Ouyang, Xiangting Chen, and Yibin Liu. Writing the original draft preparation: Xin Ouyang and Xiangting Chen. Writing, review, and editing: Bowen Liu, Yibin Liu, and Pengcheng He. Investigation and visualization: Qingbin Li, Xuhui Lin, Kailun Qiu, and Ruowen Zhao. Supervision: Pengcheng He.

## Ethics Statement

This study was based on a publicly available database and did not include any individual‐level identifying information. Ethics review was waived according to the policy of the Ethics Committee of Guangdong Provincial People's Hospital. This research utilized all publicly available data and thus met the requirements for exemption from ethical review.

## Conflicts of Interest

The authors declare no conflicts of interest.

## Supporting information


Supporting File 1

Supporting File 2

## Data Availability

Data used in this study are available from the Global Burden of Disease Results Tool (https://vizhub.healthdata.org/gbd-results/). The R code used for this analysis is available from the corresponding author on reasonable request and will be deposited in a public repository upon acceptance.

## References

[1] R. Su , W. Ze , R. Huang , Y. Guo , G. Liu , and B. Zhang , “Global Ischemic Heart Disease Burden Attributable to Environmental Risk Factors, 1990‐2021: An Age‐Period‐Cohort Analysis,” Frontiers in Public Health 13 (2025): 1622108, 10.3389/fpubh.2025.1622108. 40823230 PMC12354537

[2] A. Singh , B. Collins , A. Qamar , et al., “Study of Young Patients With Myocardial Infarction: Design and Rationale of the YOUNG‐MI Registry,” Clinical Cardiology 40 (2017): 955–961, 10.1002/clc.22774. 28805969 PMC5761653

[3] X. Liu , Y. Wu , F. Li , et al., “Global Burden of Early‐Onset Ischemic Heart Disease, 1990 to 2019,” JACC: Advances 4 (2025): 101466, 10.1016/j.jacadv.2024.101466. 39811756 PMC11731480

[4] C. Andersson and R. S. Vasan , “Epidemiology of Cardiovascular Disease in Young Individuals,” Nature Reviews Cardiology 15 (2018): 230–240, 10.1038/nrcardio.2017.154. 29022571

[5] H. Dai , A. A. Much , E. Maor , et al., “Global, Regional, and National Burden of Ischaemic Heart Disease and Its Attributable Risk Factors, 1990‐2017: Results From the Global Burden of Disease Study 2017,” European Heart Journal ‐ Quality of Care and Clinical Outcomes 8 (2022): 50–60, 10.1093/ehjqcco/qcaa076. 33017008 PMC8728029

[6] J. Chung‐Hall , L. Craig , S. Gravely , N. Sansone , and G. T. Fong , “Impact of the WHO FCTC over the First Decade: A Global Evidence Review Prepared for the Impact Assessment Expert Group,” Tobacco Control 28 (2019): s119–s128, 10.1136/tobaccocontrol-2018-054389. 29880598 PMC6589489

[7] Z. Su , Z. Zou , S. I. Hay , et al., “Global, Regional, and National Time Trends in Mortality for Congenital Heart Disease, 1990–2019: An Age‐Period‐Cohort Analysis for the Global Burden of Disease 2019 Study,” eClinicalMedicine 43 (2022): 101249, 10.1016/j.eclinm.2021.101249. 35059612 PMC8760503

[8] M. T. Lee , D. Mahtta , D. J. Ramsey , et al., “Sex‐Related Disparities in Cardiovascular Health Care Among Patients With Premature Atherosclerotic Cardiovascular Disease,” JAMA Cardiology 6 (2021): 782–790, 10.1001/jamacardio.2021.0683. 33881448 PMC8060887

[9] S. Tiwari , O. Løvsletten , B. K. Jacobsen , et al., “Occasional Smoking Is a Risk Factor for Myocardial Infarction in the Population‐Based Tromsø Study, 2001–21,” European Journal of Preventive Cardiology, ahead of print (2025): zwaf182, 10.1093/eurjpc/zwaf182. 40146252

[10] C. J. Murray , “Quantifying the Burden of Disease: The Technical Basis for Disability‐Adjusted Life Years,” Bulletin of the World Health Organization 72 (1994): 429–445. 8062401 PMC2486718

[11] A. D. Lopez and C. C. J. L. Murray , “The Global Burden of Disease, 1990–2020,” Nature Medicine 4 (1998): 1241–1243, 10.1038/3218. 9809543

[12] GBD 2023 Disease and Injury and Risk Factor Collaborators , “Burden of 375 Diseases and Injuries, Risk‐Attributable Burden of 88 Risk Factors, and Healthy Life Expectancy in 204 Countries and Territories, Including 660 Subnational Locations, 1990–2023: A Systematic Analysis for the Global Burden of Disease Study 2023,” Lancet 406 (2025): 1873–1922, 10.1016/S0140-6736(25)01637-X. 41092926 PMC12535840

[13] D. T. Levy , Z. Yuan , Y. Luo , and D. Mays , “Seven Years of Progress in Tobacco Control: An Evaluation of the Effect of Nations Meeting the Highest Level MPOWER Measures Between 2007 and 2014,” Tobacco Control 27 (2018): 50–57, 10.1136/tobaccocontrol-2016-053381. 27956650 PMC5966723

[14] A. Palali and J. C. van Ours , “The Impact of Tobacco Control Policies on Smoking Initiation in Eleven European Countries,” European Journal of Health Economics 20 (2019): 1287–1301, 10.1007/s10198-019-01090-x. PMC6856042 31485831

[15] H. Hiilamo and S. Glantz , “Global Implementation of Tobacco Demand Reduction Measures Specified in Framework Convention on Tobacco Control,” Nicotine & Tobacco Research 24 (2022): 503–510, 10.1093/ntr/ntab216. 34661672 PMC8887591

[16] A. Grainger Gasser , C. Welch , M. Arora , et al., “Reducing Cardiovascular Mortality Through Tobacco Control: A World Heart Federation Roadmap,” Global Heart 10 (2015): 123, 10.1016/j.gheart.2015.04.007. 26213299

[17] S. Gravely , G. A. Giovino , L. Craig , et al., “Implementation of Key Demand‐Reduction Measures of the WHO Framework Convention on Tobacco Control and Change in Smoking Prevalence in 126 Countries: An Association Study,” Lancet Public Health 2 (2017): e166–e174, 10.1016/S2468-2667(17)30045-2. 29253448

[18] G. A. Mensah , G. S. Wei , P. D. Sorlie , et al., “Decline in Cardiovascular Mortality: Possible Causes and Implications,” Circulation Research 120 (2017): 366–380, 10.1161/CIRCRESAHA.116.309115. 28104770 PMC5268076

[19] E. S. Ford , U. A. Ajani , J. B. Croft , et al., “Explaining the Decrease in U.S. Deaths From Coronary Disease, 1980–2000,” New England Journal of Medicine 356 (2007): 2388–2398, 10.1056/NEJMsa053935. 17554120

[20] S. Akter , M. R. Islam , M. M. Rahman , et al., “Evaluation of Population‐Level Tobacco Control Interventions and Health Outcomes: A Systematic Review and Meta‐Analysis,” JAMA Network Open 6 (2023): e2322341, 10.1001/jamanetworkopen.2023.22341. 37418258 PMC10329215

[21] G. Heydari , F. Chamyani , M. Masjedi , and L. Fadaizadeh , “Comparison of Tobacco Control Programs Worldwide: A Quantitative Analysis of the 2015 World Health Organization MPOWER Report,” International Journal of Preventive Medicine 7 (2016): 127, 10.4103/2008-7802.195562. 28105292 PMC5200974

[22] Y. Lee , S. Kim , M. K. Kim , I. Kawachi , and J. Oh , “Association Between Tobacco Industry Interference Index (TIII) and MPOWER Measures and Adult Daily Smoking Prevalence Rate in 30 Countries,” Globalization and Health 20 (2024): 6, 10.1186/s12992-023-01003-x. 38172937 PMC10765652

[23] M. A. Harrison , A. F. A. Marfo , A. Annan , and D. N. A. Ankrah , “Access to Cardiovascular Medicines in Low‐ and Middle‐Income Countries: A Mini Review,” Global Health Research and Policy 8 (2023): 17, 10.1186/s41256-023-00301-6. 37221559 PMC10204246

[24] M. B. Reitsma , P. J. Kendrick , E. Ababneh , et al., “Spatial, Temporal, and Demographic Patterns in Prevalence of Smoking Tobacco Use and Attributable Disease Burden in 204 Countries and Territories, 1990–2019: A Systematic Analysis From the Global Burden of Disease Study 2019,” Lancet 397 (2021): 2337–2360, 10.1016/S0140-6736(21)01169-7. 34051883 PMC8223261

[25] C. K. Chow , T. N. Nguyen , S. Marschner , et al., “Availability and Affordability of Medicines and Cardiovascular Outcomes in 21 High‐Income, Middle‐Income and Low‐Income Countries,” BMJ Global Health 5 (2020): e002640, 10.1136/bmjgh-2020-002640. 33148540 PMC7640501

[26] M. E. Cornelius , C. G. Loretan , A. Jamal , et al., “Tobacco Product Use Among Adults ‐ United States, 2021,” MMWR. Morbidity and Mortality Weekly Report 72 (2023): 475–483, 10.15585/mmwr.mm7218a1. 37141154 PMC10168602

[27] Y. Selvamani , J. Pradhan , and J. H. Fong , “Tobacco Use, Food Insecurity, and Low BMI in India's Older Population,” Nutrients 16 (2024): 3649, 10.3390/nu16213649. 39519481 PMC11547918

[28] R. R. Huxley and M. Woodward , “Cigarette Smoking as a Risk Factor for Coronary Heart Disease in Women Compared With Men: A Systematic Review and Meta‐Analysis of Prospective Cohort Studies,” Lancet 378 (2011): 1297–1305, 10.1016/S0140-6736(11)60781-2. 21839503

[29] L. S. Flor , J. A. Anderson , N. Ahmad , et al., “Health Effects Associated With Exposure to Secondhand Smoke: A Burden of Proof Study,” Nature Medicine 30 (2024): 149–167, 10.1038/s41591-023-02743-4. PMC10803272 38195750

[30] M. Teramoto , H. Iso , I. Muraki , K. Shirai , and A. Tamakoshi , “Secondhand Smoke Exposure in Childhood and Mortality From Coronary Heart Disease in Adulthood: The Japan Collaborative Cohort Study for Evaluation of Cancer Risk,” Journal of Atherosclerosis and Thrombosis 30 (2023): 863–870, 10.5551/jat.63857. 36261366 PMC10406645

[31] D. C. López‐Medina , C. Candal‐Pedreira , J. Rey‐Brandariz , et al., “Evolution and Characteristics of Studies Estimating Attributable Mortality to Second‐Hand Smoke: A Systematic Review,” European Journal of Public Health 34 (2024): 557–565, 10.1093/eurpub/ckae049. 38531674 PMC11161163

[32] M. J. Mears , H. L. Hookfin , P. Bandaru , P. Vidal , K. I. Stanford , and L. E. Wold , “Electronic Nicotine Delivery Systems and Cardiovascular/Cardiometabolic Health,” Circulation Research 132 (2023): 1168–1180, 10.1161/CIRCRESAHA.123.321565. 37104558 PMC10154046

[33] A. Tansawet , T. Anothaisintawee , S. W. Boonmanunt , et al., “Electronic Cigarettes and Cardiovascular Diseases: An Updated Systematic Review and Network Meta‐Analysis,” Tobacco Induced Diseases 23 (2025): 208065, 10.18332/tid/208065. PMC12412302 40917139

[34] J. J. Rose , S. Krishnan‐Sarin , V. J. Exil , et al., “Cardiopulmonary Impact of Electronic Cigarettes and Vaping Products: A Scientific Statement From the American Heart Association,” Circulation 148 (2023): 703–728, 10.1161/CIR.0000000000001160. 37458106

