# Supplementary material for: Tobacco‐Attributable Burden of Early‐Onset Ischemic Heart Disease From 1990 to 2023: A Global Analysis Using GBD 2023 and Age‐Period‐Cohort Modeling
Source: Clin Cardiol. 2026 Aug 24;49(8):e70451. doi: 10.1002/clc.70451 (PMC13501498; doi:10.1002/clc.70451)
Supplement: Supplementary file 1 [file CLC-49-e70451-s001.docx]

**Supplementary Materials**

The following supporting information is provided separately: Figure S1, APC estimates for tobacco-attributable DALYs in males; Figure S2, APC estimates for tobacco-attributable DALYs in females; Figure S3, APC estimates for tobacco-attributable deaths in males; Figure S4, APC estimates for tobacco-attributable deaths in females; Table S1, sensitivity analysis comparing males and females within the common age range of 40-54 years.

Figure S1, APC estimates for tobacco-attributable DALYs in males;

Figure S2, APC estimates for tobacco-attributable DALYs in females;

Figure S3, APC estimates for tobacco-attributable deaths in males;

Figure S4, APC estimates for tobacco-attributable deaths in females;
